# Shape-memory sawtooth-arm embracing clamp used in complex femoral revision hip arthroplasty for stem stability: average 9-year follow-up study

**DOI:** 10.1186/s12891-023-07080-8

**Published:** 2023-12-15

**Authors:** Yi Hu, Zhengquan Xu, Hua Qiao, Keyu Kong, Huiwu Li, Jingwei Zhang

**Affiliations:** 1 Shanghai Key Laboratory of Orthopaedic Implants, Department of Orthopaedic Surgery, Shanghai Ninth People’s Hospital, Shanghai Jiaotong University School of Medicine, 639# Zhizaoju Road, Shanghai, 200011 P. R. China; 2 Department of Orthopaedic Surgery, Suzhou Hospital, Nanjing Medical University, 16# Baita West Road, Suzhou, Jiangsu 215000 P. R. China

**Keywords:** Shape-memory sawtooth-arm embracing clamp, Revision hip arthroplasty, Femoral revision, Mid to long-term follow-up

## Abstract

**Background:**

Nickel-Titanium shape-memory sawtooth-arm embracing clamps (SSECs) have been used in revision total hip arthroplasties (rTHAs) to protect stem stability. This study was to introduce this technique and report its mid to long-term clinical and radiographic outcomes.

**Methods:**

We retrospectively reviewed all patients implanted with SSECs in our department from January 2008 to December 2015. 41 patients (41 hips) were finally included. Radiographs and Harris hip scores (HHS) were collected. Radiographs were blindly analyzed for evidence of loosening, subsidence and stress shielding. HHS were compared to previous records by student’s t tests. The average follow-up period was 9.3 years.

**Results:**

All stems were stably fixed with no signs of loosening. The mean stem subsidence was 0.9 mm (range, 0 to 3 mm). Only one patient (2.4%) demonstrated the fourth degree of stress shielding, with the others none or minor bone resorption. The mean HHS at the final follow-up was 84.2 (range, 81 to 91), which was improved from 17.4 (range, 0 to 37) before surgery. No implant failures or re-revisions occurred. Dislocation occurred in 1 case during the follow-up period.

**Conclusions:**

The SSEC protected stem fixation and achieved favorable clinical and radiographic outcomes in this 9-year follow-up study. It offered an additional extramedullary fixation option for surgeons to choose from in treating complex femoral revision arthroplasties.

## Introduction

The frequency of revision total hip arthroplasty (rTHA) is projected to grow to over 85,000 by 2030 in the United States, which places a heavy economic burden on the healthcare system [1]. In these cases, over 50% of femoral components has to be revised [2].

Femoral revision is often complicated by mild or advanced bone loss, or the poor integrity of the remaining bone stock, which greatly impairs implant stability [3]. For this reason, proximally porous-coated or modular femoral components, or extensively porous-coated stems, or even modular tapered fluted stems are frequently selected in femoral revisions. Besides, in these cases, extramedullary fixation techniques, including cables or steel wires, plates or locking compression plates and strut cortical allografts, are always supplemented to protect the solid primary stability of the cementless stems in surgery, which have shown satisfactory clinical results [4–7]. However, there are reported drawbacks to their use. Fixation with prophylactic wires was not always reliable in stopping a femoral crack from propagating, and the force required to form a crack was not reduced if cables were used improperly [8]. Cable-plate system performed poorly in fixation of the trochanteric fractures in rTHAs, with nonunion rate of 31.4%, wire breakage of 28.6% and re-revision of 14.3% [9]. Although strut cortical allografts could restore bone stock and have the potential to reattach host soft tissues, they might transmit diseases and arise immune rejection [10]. In addition, it is difficult to obtain appropriate allografts, and it also brings higher technical demands, risks of nonunion or graft resorption [11].

**Fig. 1:**
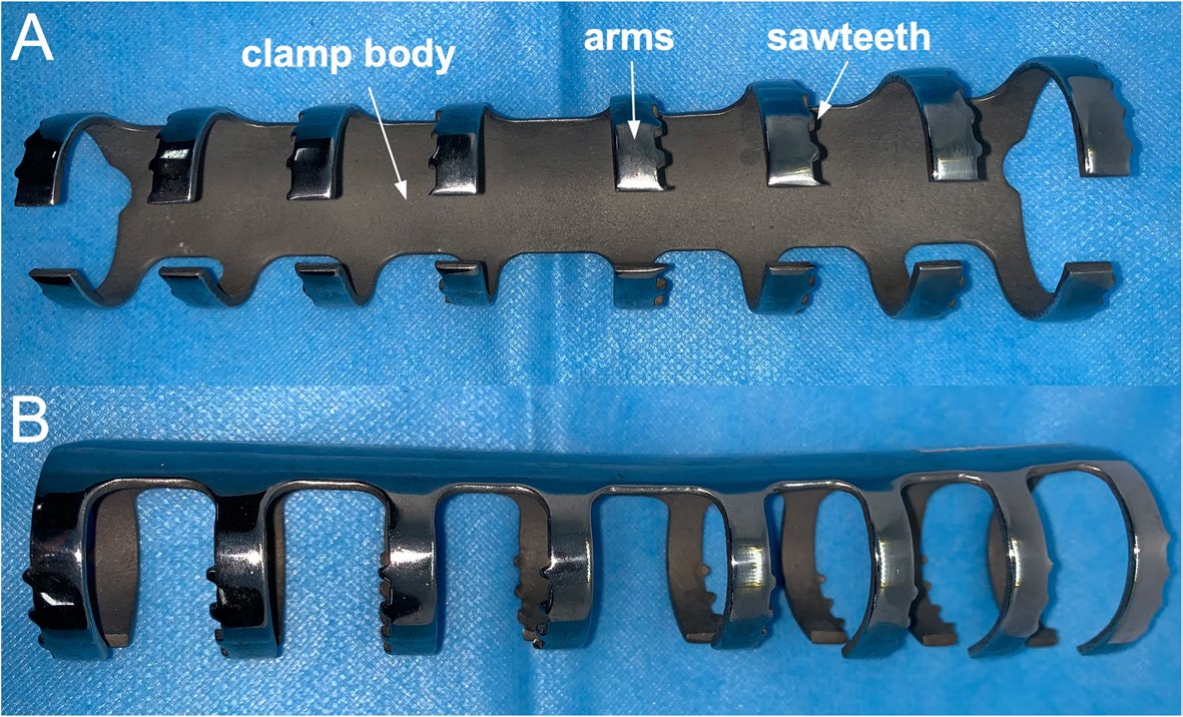
**A** The front view of the SSEC. It is consisted of three parts: a clamp body, pairs of arms and sawteeth. The clamp body is to be placed on the tension side of the femur. Pairs of arms are designed to embrace around the femoral diaphysis symmetrically, and the sawteeth protrudre internally to the axis of the bone. **B** The lateral view of the SSEC

In this study, we provided an additional option for extramedullary fixation. We have been using nickel-titanium shape-memory sawtooth-arm embracing clamps (Ni-Ti SSECs; Si-Ai Hi-Tech Ltd, Shanghai, China) to grip the femoral diaphysis for protecting stem fixation in femoral revisions, since they were invented in 1990s [12]. This device includes a clamp body on the tension side of the femur, pairs of arms embracing around and fishhook-like sawteeth protruding internally (Fig. 1). The shape and stiffness of this nickel-titanium alloy is temperature sensitive by means of the Martensite-Austenite transformation. When cooled down at 0 to 4℃, the alloy is in the Martensite phase, and allows easy bending and shaping. When warmed up to about 40℃, it changes back to the Austenite phase with the original shape and stiffness restored [12]. By this speciality, the cooled SSEC could be inserted easily, and afterwards when warmed up, embracing arms returned to the pre-designed shapes which grip the femoral diaphysis firmly and offer additional fixation force. It eased surgical manipulation, reduced muscle stripping and achieved excellent clinical outcomes in femoral revision arthroplasties at about 4 years after surgery [13].

The SSEC is a extramedullary device, which functions through supporting by the body, and clamping and fixation by the arms and sawteeth. Although so far, welcome short to mid-term clinical outcomes have been shown, which were mentioned above, concerns on its actual long-term effects still existed [13–15]. Since auxiliary clamping body and arms supported bone growth, stress shielding and consequent osteoporosis become a common concern, especially in a longer follow-up period. Besides, it remains questionable whether the SSEC really benefits long-term implant durability or itself could survive through postoperative daily activities without loosening or breakage. In cases using longer clamps, femoral anterior bow matters and thigh pain might also happen. Such problems deserve attention.

Hence, in this study, we aimed to further investigate the mid to long-term clinical and radiographic outcomes of this technique. We enrolled 41 patients, in whom SSECs were implanted during femoral revision arthroplasty, and emphasized on their stress shielding effect, the stability and survivorship of the stem and clamp, and patients’ functional scores after average 9-year after surgery. We hypothesized that Ni-Ti SSECs was a reliable extramedullary fixation technique, which achieved little stress shielding, stable fixation and favorable clinical scores in mid to long-term follow-up.

## Methods

### Patients

This study was approved by the Ethics Committee of our hospital. We searched our medical record registry to identify all patients in whom embracing clamps were implanted at the time of rTHA for femoral prosthesis loosening or osteolysis between January 2008 (when the registry system was first used) and December 2015. The designed minimum follow-up was 6 years. Patients diagnosed with periprosthetic fractures or bone tumour, or for acetabular revisions were excluded. A total of 46 patients (46 hips) were available for this study and were retrospectively reviewed. Four patients were lost to follow-up (one patient at 3 months, one at 3 years, and two at 4 years) and one patient died from hip-unrelated causes within 4 years. Above patients were excluded. Accordingly, the remaining 41 patients (41 hips, 12 males and 29 females) were finally included into this retrospective study. These patients received anteroposterior and lateral radiographs (RAX, Siemens, Erlangen, Germany) for the pelvic and affected femur, and Harris hip scores (HHS) evaluation at the final follow-up. The mean age of them was 74.3 years (range, 33.2 to 84.4 years), and the mean height, weight and BMI was 1.63 m (range, 1.55 to 1.75 m), 57 kg (range, 42 to 76 kg) and 21.5 kg/m^2^ (range, 17.5 to 24.8 kg/m^2^), respectively. The indication for femoral revision was all aseptic loosening. Based on preoperative radiology and confirmation in surgery after removing the implant, 19 patients presented with Type IIIA defects, 18 with Type IIIB defects, and four with Type IV defects by the Paprosky classification [16]. Twenty-five patients underwent concomitant acetabular revision secondary to loosening at the time of femoral component revision. The indication for using SSEC is whenever the stem stability might be endangered and additional protection is needed in any situations. It was used for various procedures: 12 cases to protect stem fixation where bone was severely defected, 11 cases to prevent femoral fracture where long cortical windows were created for extracting cement fragments, 9 cases to fix the femoral shaft wedge osteotomy and 9 cases to fix the extended trochanteric osteotomy. The average follow-up period after rTHA was 9.3 years, ranging from 6.9 to 14.6 years.

### Surgery procedures and SSECs

Surgeries were performed by the same group of qualified and experienced surgeons in posterolateral approach. The femoral stems were all revised in these cases. After removing the failed implant, interface membranes in the femoral canal were cleared. Fluted, modular, tapered revision stems (Lima-Lto, Udine, Italy) were inserted in all cases.

**Fig. 2:**
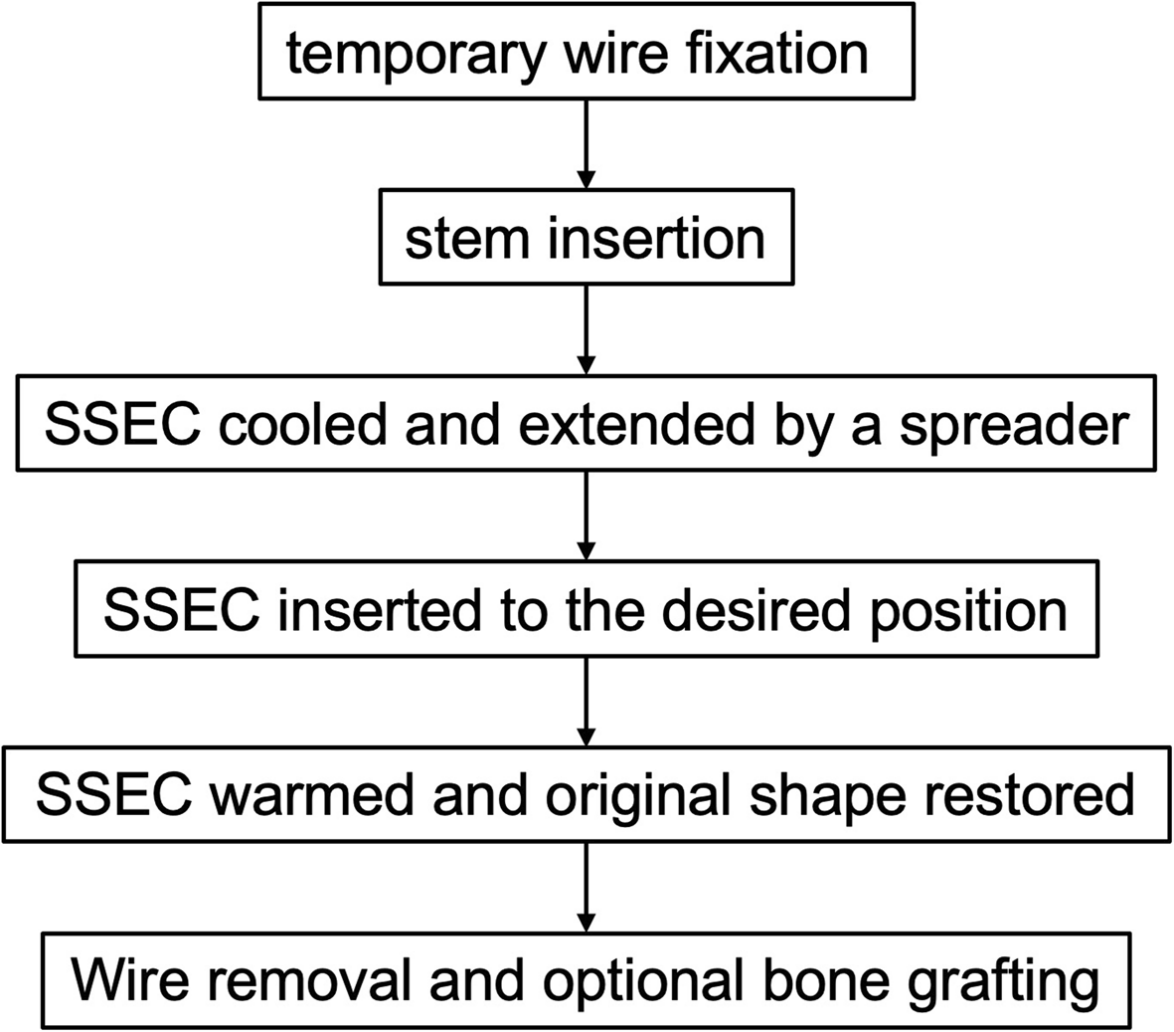
The usage process of the SSEC

Then, SSEC was implanted. The flowchart of SSEC usage was shown in Fig. 2. Usually, wires or cables were first encircled around for temporary fixation to prevent intraoperative cracks propagating after initial reduction. Then, the revision stem was inserted. The SSEC selected for surgery had a diameter of 10 to 20% smaller than that of the treated femur, and a length extending over three pairs of arms on each side of the unstable line. After submerged in iced sterile saline for 3 to 5 min, arms of the clamp could be extended by a spreader forcep. Then the SSEC was inserted with its body on the tension side. Once the proper position was ensured, it was warmed up by hot saline (37℃). After about 5 min, it restored to its original shape, holding the defected femur and stem firmly. If wires or cables were difficult to remove after final fixation, they were retained.

Optional bone grafting could be selected, while none was performed in this cohort of patients. The intraoperative fixtion was all stable when testing extremity movements.

### Rehabilitation

Postoperative rehabilitation was personally designed, based on surgical treatments, the stability of final fixation and patients’ physical condition. Generally, early movements were encouraged, and patients were allowed to sit on the first day after surgery. Partial weight-bearing, as tolerated, was allowed in the succeeding 6 weeks. Depending on postoperative radiographic evaluation, gradual full weight-bearing was permitted at 8–12 weeks after surgery.

### Evaluation

All patients were asked to follow postoperative clinical and radiographic examinations immediately after surgery, at 6 weeks, 3 months, 6 months and then annually. Radiographs of these patients before and after surgery and at the last follow-up were collected, and blindly reviewed by three of the authors. Below radiologic features were evaluated, and results were recorded following only patient identification numbers. Femoral component stability was evaluated with the criteria by Engh et al [17]. Stable bone ingrowth was defined as no subsidence, and no or minimal radiopaque line formation around the stem, while unstability was defined as definite progressive subsidence or migration, or at least divergent radiopaque lines partially surrounding [17]. Femoral component subsidence was measured as the change in distance between the center of femoral head and the most proximal point on the lesser trochanter. When the lesser trochanter became unavailable, the tip of the greater trochanter was served as alternative [18]. Trochanter stress shielding was graded into four degrees, from only the proximal medial edge of the cut femoral neck rounded off to severe cortial resorption extended into the diaphysis [17]. Clinical outcomes for each patient before surgery and at the final follow-up were examined in the HHS, and the HHS at different postoperative stages were compared [19]. Any SSEC retrieval for mechanical failure, infection, immune response or any other reasons, was also recorded.

### Statistical analysis

Two-sided student’s t tests were performed to evaluate the statistical significance of postoperative HHS improvements. *p* values of < 0.05 were considered significant. The analysis was all conducted by SPSS for Mac (version 26.0; SPSS, Chicago, IL, USA).

## Results

### Radiographic discoveries

**Fig. 3:**
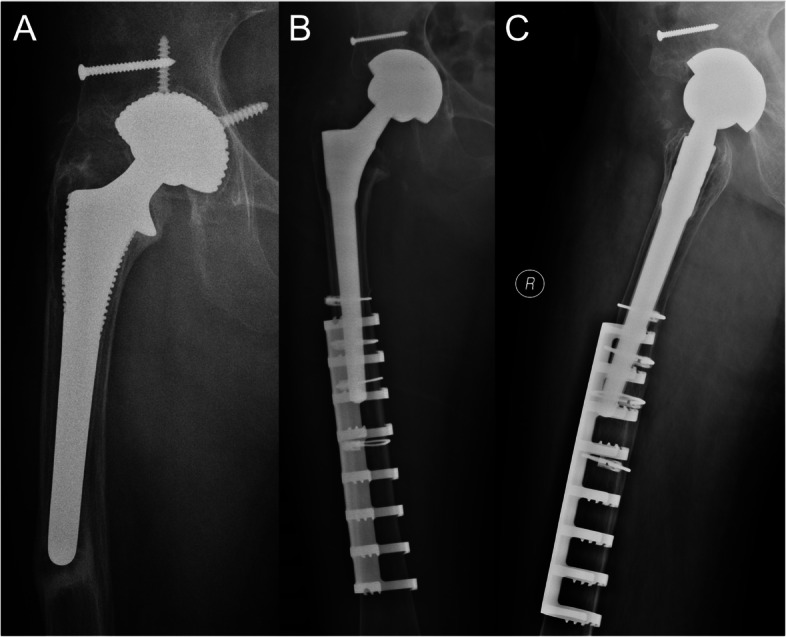
A 77-year-old female patient developed right aseptic loosening 15 years after primary hip arthroplasty. **A** Preoperative radiograph. **B** Immediately after surgery. The acetabulum and femur was revised, and a SSEC was inserted. **C** 10 years after surgery, the stem was stably fixed and there was no evident bone resorption around the proximal femur

**Fig. 4:**
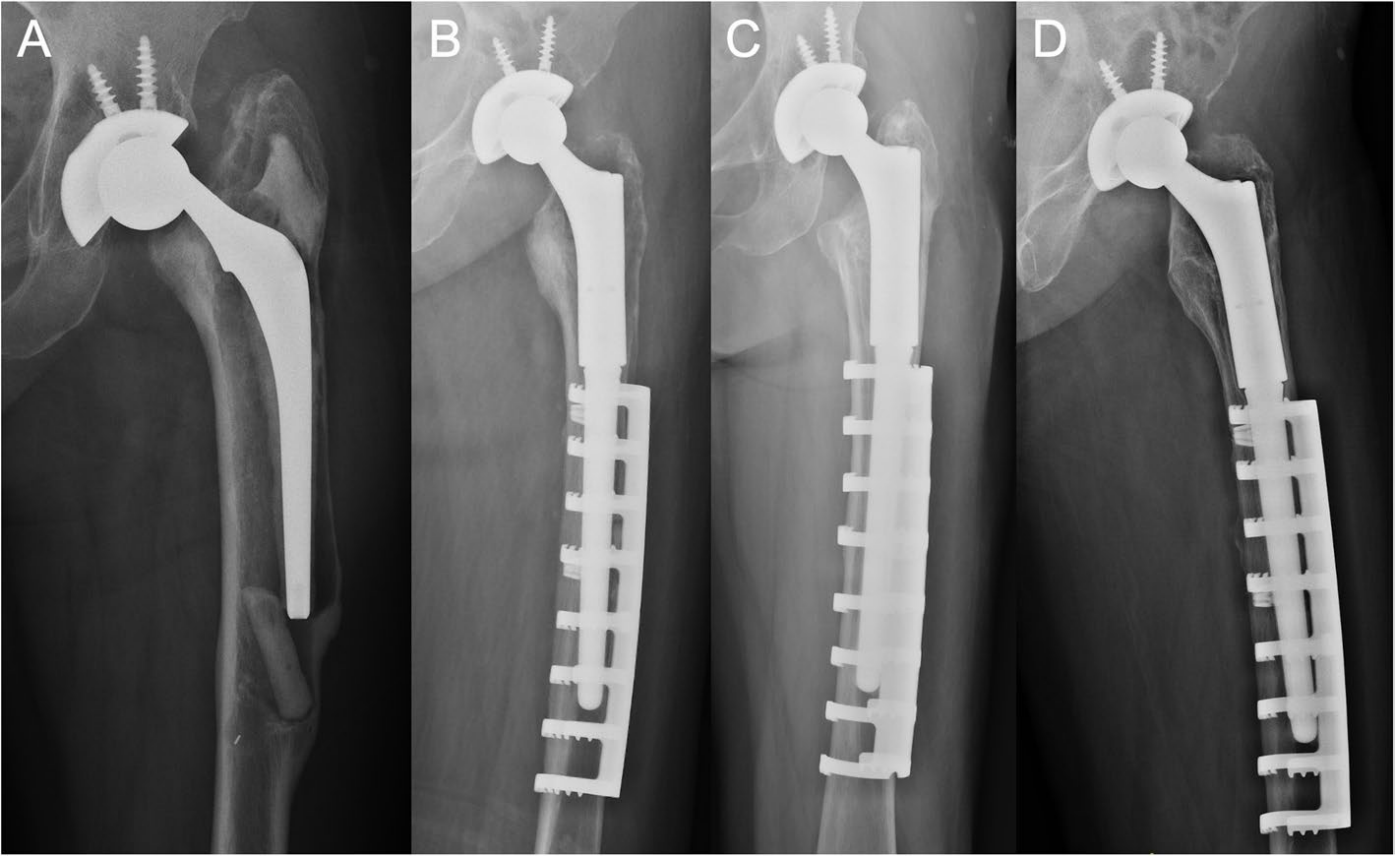
A 71-year-old female patient experienced left aseptic loosening 10 years after primary hip arthroplasty. **A** Preoperative radiograph. **B** Immediately after surgery. **C** 3 years after surgery. No evident stress shielding was detected. **D** 8 years after surgery, there was obvious cortical and diaphyseal bone resorption in the proximal medial femur

Based on the last follow-up radiography, no progressive radiopaque line was found surrounding the cementless femoral stem, demonstrating well bone ingrowth fixation and no signs of radiological loosening (Fig. 3). All stems but one were in neutral alignments (97.6%). The mean stem subsidence was 0.9 mm (range, 0 to 3 mm). 40 patients demonstrated none or minor host resorption around the proximal medial edge of the femoral neck, which was close to the first degree of stress shielding. Obvious cortical and diaphyseal bone resorption in the proximal medial femur was detected in only one case (2.4%), which was categorized into the fourth degree of stress shielding (Fig. 4). All SSECs were locked in position with no signs of lossening, displacement or breakage, when compared to previous series of radiographies.

### The HHS results and Complications

These patients got an average HHS of 17.4 (range, 0 to 37) before surgery, which was significantly improved to a mean of 79.1 (*p* < 0.01; range, 62 to 89) at 4 years postoperatively. This tendency was maintained in this average 9-year follow-up study, achieving an average of 84.2 (range, 81 to 91). However, when compared to the previous 4 years’ scores, this improvement was of no statistical significance (*p* > 0.05). When considering discrete HHS pain or functional scores, the average pain score was 40.5 (range, 40 to 44), and the average functional score was 43.8 (range, 41 to 47) at the final examination. While at the 4-year time, the average pain and functional scores were 39.4 (range, 30 to 44) and 39.8 (range, 32 to 45), respectively. It showed that similar pain scores were achieved at both 4 and 9 years, and functional progression contributed to the total score elevation. No significant motion restriction had been apparent since half a year after surgery, with over 90° of flexion in all hips. By 12 months, all patients were able to wear shoes and navigate floors and stairs. At about 2 years after surgery, all patients could walk without assistance or with a cane for the sake of safety. No obvious thigh pain was reported.

None of these patients underwent any re-revision arthroplasties. Only one dislocation occurred (2.4%), which was managed with closed reduction and skin traction. No SSECs were retrieved finally for mechanical failure, immune response, or any other reasons in this mid to long-term follow-up study of average 9.3 years.

## Discussion

The current study showed favourable mid to long-term clinical and radiographic discoveries in using Ni-Ti SSECs for additional femur and stem fixation protection at about 9 years after rTHAs. Specifically, no signs of prosthetic radiological loosening were detected. Over 95% of patients demonstrated none or minor stress shielding effects. All SSECs survived the follow-up period, and no patient received any re-revision arthroplasties for any reasons at the final examination.

In femoral revision arthroplasties, especially cases with severe diaphyseal loss, extensively porous-coated, or modular tapered, or other kinds of revision stems were frequently used under precise indications. Sometimes, it may be necessary to restore the bone stock for component rotational and axial stability. Due to lack of space for screws around the stem, extramedullary fixation techniques, including cables or wires, plates or locking compression plates and strut cortical allografts, are always served as supplements in these cases for further protecting deficient femur and stem fixation. Single cerclage cabling could achieve enough stability and reliable outcomes for intraoperative calcar cracks in primary THA with cementless tapered wedge stems [20]. However, when the femoral isthmus available for press-fit of the stem was less than 2 to 4 cm or in osteotomy cases, cerclage wires becomes unreliable and a more solid fixator is necessary [21]. Cable-plate systems and strut cortical allografts are common alternatives in such situations for more rigid fixation. Wallace et al. reported that extensively porous-coated stems combined with augmentation devices, including trochanteric plate and cabling systems and cortical strut allografts, had excellent long-term clinical outcomes of about 10 years in rTHAs with 98% of stems achieving stable bony ingrowth and no mechanical failures or re-revions [22]. In such cases, modular tapered titanium stems have gained more popularity by offering surgical versatility and increasing proximal fixation. Besides, a plate-cable system has drawbacks including relatively complex manipulations and high bone nonunion rate which was over 30% in trochanteric fixation [9]. Fresh-frozen unprocessed allografts were also associated with risks of viral transmission of approximately 1 in 500,000 and secondary bacterial infection [23].

In this study, we provided an additional option for extramedullary fixation, and explored its mid to long-term clinical outcomes. The SSEC, first invented in 1990s by Dai et al., was designed as a kind of extramedullary fixator [12]. It is consisted of three components: the body, arms and sawteeth [12]. The body and arms form two-thirds of its circumference on the cross-section, which clamp the femoral diaphysis outside and increases its anti-bending effects. The arms’ ends are bent more medially, after exceeding the semi-circle, to better match the shape of long bone on the transverse plan. In the axial direction, each sawtooth protrudes internally like a triangle, with the base on the arms and vertex against the femur. The pression and friction from the vertex offered anti-torsion effects. Above biomechanical effects had already been proved in vitro and in vivo in dogs that SSECs’ anti-bending and anti-torsion strength was analogous to those of commonly used plates [24].

The SSEC was initially used in long bone fractures in clinics, which offered stable fixation and allowed early rehabilitation [24]. Zhao et al. applied it in Vancouver type B1 and type C periprosthetic femoral fractures, and found that it contributed to bone union and no implant failures or malunions occurred [14]. Li et al. expanded its application to rTHAs, and detected that it effectively promoted femoral osteotomy healing and protected against femoral fracture [13]. All stems were stably fixed with no signs of mechanical failure 4 years after surgery [13]. In this study, we further proved its validity to mid to long-term that revision stems were reliably fixed, together with SSECs, survived average 9 years’ postoperative period without lossening, displacements or other kinds of mechanical failure. Further studies were needed for long-term clinical outcomes with SSECs.

When using extramedullary fixators, like plates, allografts and SSECs, concerns of stress shielding and thigh pain, which is the outcome of component modulus mismatch, are quite common. In this study, only one patient (2.4%) demonstrated fourth degree of stress shielding, with the others none or minor host bone resorption, suggesting low stress shielding effects with SSECs. It could be probably explained from the biomechanical view. First, fishhook-like and face-to-face design of the sawteeth only prevent fracture distraction, but allow axial compression by gravity, which favors bone healing and reduces stress shielding. Second, the anti-loading effect of SSEC’s body was significantly lower than that of rigid plates [25]. Third, SSECs are made of biocompatible Ni-Ti alloy and have an elastic modulus of 54GPa, which is close to that of cortical bone [26]. By these characteristics, host bone probably beared more weights, and consequently, there was less bone resorption and stress shielding effects. Krishnamurthy et al. reported pronounced stress shielding with extensively coated components in 6% of patients at about 8 years after surgery [27]. Although our result showed only 2.4% of patients with obvious stress shielding, so many confounding variables existed when comparing to other studies, and it was hard to draw any conclusions.

In some complex femoral revisions, cortical strut allografts were applied to augment bony deficiency and provide extra fixation. Outstanding advantages of this technique over other rigid fixation involve lower stress shielding and the ability to restore bone stock [28]. Although favourable clinical outcomes were achieved, it still has several drawbacks. As a kind of biomaterial, it is rare and expensive, and would potentially arouse immune rejection response [10]. The surgical technique is demanding, which requires more vascular muscle stripping and longer procedure time. Postoperative allograft resorption might also affect prosthesis longevity [29]. In this study, we found SSEC also had such advantages but eliminated the defects. Ni-Ti alloy is relative cheaper, and allows wider manufacture and clinical application. Besides, Ni-Ti alloy has been proved to have great biocompatibility with low risk of immune response [30]. As for manipulation, SSEC could be inserted into the muscle fiber gap, which eases the procedure and reduces stripping extent. However, to be noted, the SSEC also has its shortcomings. First, it cannot restore bone quantity. Additional bone grafting must be performed when necessary. Second, there is time limits for insertion. Once the SSEC is warmed back, it is difficult to adjust position again, while we did not encounter this situations in our practice. As for removal, the procedure is just reverse to the insertion: after cooled down by iced sterile saline, the SSEC became soft, and could be bent and taken out. Nevertheless, we only retrieved one SSEC following the patient’s requesting. In this case, the SSEC was initially inserted to fix the femoral shaft fracture, and was taken out one year after surgery smoothly and successfully when the fracture was union. Thus, it was not included in this study, and we just reported it as a kind of clinical experience.

The results of the current study need to be interpreted in light of several limitations. First, the sample size in this study was relatively small. Only 41 patients from a single center were included, and the results might not be robust. However, considering that complex femoral revision cases are relatively rare and few hospitals used the technique of SSECs, we still chose to present the outcome of this technique and our experience. The conclusions need to be confirmed by researches of a larger sample size in the future. Second, there was no control group. In this study, we introduced the SSEC as an additional option for extramedullary fixation, and further proved its reliable clinical outcomes in rTHAs to the mid to long-term. We did not intend to compare the SSEC with other kinds of extramedullary fixation techniques, like cables, plates or cortical allograft struts, in this study. We only introduced the SSEC and reported its mid to long-term outcomes, and the lack of control group did not influence our conclusions. Further well conducted controlled studies were needed to explore the advantages and disadvantages among different extramedullary fixation options.

## Conclusion

The SSEC used in complex femoral revisions offered reliable mid to long-term clinical and radiographic follow-up outcomes in protecting the fixation of stems and kinds of osteotomy after average 9.3 years after surgery. It is an effective and reliable extramedullary fixation technique for surgeons to choose from when dealing with complex femoral revision arthroplasties.

## Data Availability

The raw data supporting the conclusions of this article will be made available by the corresponding author (JWZ), without undue reservation, to any qualified researcher.

## Abbreviations

HHS: Harris hip scores
Ni-Ti SSEC: Nickel-Titanium shape-memory sawtooth-arm embracing clamp
rTHA: Revision total hip arthroplasty
